# Food Supplement Use Differs from the Recommendations in Pregnant Women: A Multinational Survey

**DOI:** 10.3390/nu14142909

**Published:** 2022-07-15

**Authors:** Ella Koivuniemi, Kathryn Hart, Natalia Mazanowska, Stefania Ruggeri, Bernadette Egan, Laura Censi, Romana Roccaldo, Lilja Mattila, Pasquale Buonocore, Eliisa Löyttyniemi, Monique M. Raats, Miroslaw Wielgos, Kirsi Laitinen

**Affiliations:** 1 Research Centre for Integrative Physiology and Pharmacology, Institute of Biomedicine, University of Turku, 20014 Turku, Finland; lilja.h.mattila@utu.fi (L.M.); kirsi.laitinen@utu.fi (K.L.); 2 Department of Nutritional Sciences, School of Biosciences and Medicine, Faculty of Health and Medical Sciences, University of Surrey, Guildford GU2 7XH, UK; k.hart@surrey.ac.uk; 3 First Department of Obstetrics and Gynecology, Medical University of Warsaw, 02-091 Warsaw, Poland; natalia.mazanowska@gmail.com (N.M.); miroslaw.wielgos@wum.edu.pl (M.W.); 4 Council for Agricultural Research and Economics (CREA), Research Centre for Food and Nutrition, 00178 Rome, Italy; stefania.ruggeri@crea.gov.it (S.R.); laura.censi@crea.gov.it (L.C.); romana.roccaldo@crea.gov.it (R.R.); pasquale.buonocore@crea.gov.it (P.B.); 5 Food, Consumer Behaviour and Health (FCBH) Research Centre, University of Surrey, Guildford GU2 7XH, UK; m.egan@surrey.ac.uk (B.E.); m.raats@surrey.ac.uk (M.M.R.); 6 Biostatistics, Department of Clinical Medicine, University of Turku, 20014 Turku, Finland; eliisa.loyttyniemi@utu.fi; 7 Functional Foods Forum, University of Turku, 20014 Turku, Finland; 8 Department of Obstetrics and Gynecology, Turku University Hospital, 20521 Turku, Finland

**Keywords:** dietary supplement, prenatal supplement, vitamin, mineral, gestation

## Abstract

The aim was to investigate, among pregnant women, (1) the use of food supplements and (2) the awareness of food supplement recommendations and beliefs about food supplement use in four European countries: Finland, Italy, Poland, and the United Kingdom. The participants (*n* = 1804) completed an online questionnaire with predefined statements. Daily intakes of vitamins and minerals were calculated using uploaded pictures or weblinks of the supplement packages. Country differences were assessed. Most participants (91%) used at least one food supplement during pregnancy. A prenatal multivitamin was the most commonly used supplement type (84% of the users), and 75% of the participants thought consumption of multivitamin is recommended. Of the participants, 81% knew that folic acid is recommended during pregnancy while 58% knew the recommendation for vitamin D. In 19% of the supplement users, the daily safe upper intake limit of at least one nutrient was exceeded. Nevertheless, most participants agreed that they knew which supplements (91%) and doses of supplements (87%) needed to be used during pregnancy. To conclude, the majority of the participants used food supplements, but lower proportions knew and adhered to the recommended intakes. Between-country differences were observed in the use and knowledge of and beliefs regarding supplements. The results suggest a need for assessment and monitoring of supplement use in antenatal care to ensure appropriate use.

## 1. Introduction

Adequate intakes of vitamins and minerals from the diet are important during pregnancy as the need for nutrients increases to support the growth and development of the fetus and increased tissue growth and metabolism of the mother [1]. Diets that align with the dietary recommendations will meet most nutritional needs during pregnancy. Nevertheless, to ensure sufficient intake of specific vitamins and minerals, recommendations for the use of particular food supplements during pregnancy have been made. The most commonly recommended food supplement for the general population of pregnant women is folic acid; however, other micronutrients such as iron and calcium are recommended as supplements either for the general population of fertile and pregnant women or for individuals with deficient intakes [2]. Setting recommendations is essential as the use of food supplements is not without risk; high doses may be teratogenic, as is the case with vitamin A [3], or potentially harmful for the health of the mother, such as iron, which has been suggested to increase the risk of gestational diabetes [4,5]. It is also of note that vitamins and minerals may alter epigenetic effects in both mothers and their offspring [6]. As defined in the European Union’s food legislation [7], food supplements are food products that differ from normal foods by either their appearance or the way in which they are used, e.g., they are in tablet, capsule, or liquid form in measured doses and do not provide significant amounts of energy.

Previous findings on food supplement use, mostly from cohort studies or clinical trials, indicate that 69–96% of pregnant women use food supplements in Western countries [8,9,10,11,12,13]. However, there is little information on the daily intakes of nutrients from food supplements and whether the doses are in line with local recommendations. Further, women’s awareness of the recommendations and possible benefits and risks of supplement use may affect their willingness to use supplements during pregnancy [14,15]. Information on the food supplement usage of pregnant women and their awareness of supplements is needed to establish and track awareness levels, thus informing the development of health initiatives that ensure the optimal intake of nutrients during pregnancy. In addition, international comparisons of the current practices and beliefs may support the harmonization of the guidelines across countries.

The objectives of this study were to, among pregnant women, investigate (1) the use of food supplements, particularly considering the recommended and safe intakes; and (2) the awareness of food supplement recommendations and beliefs about food supplement use during pregnancy in four geographically and socioeconomically diverse European countries: Finland, Italy, Poland, and the United Kingdom (UK).

## 2. Materials and Methods

### 2.1. Study Design and Study Population

This cross-sectional survey collected data on food supplement knowledge and use (including vitamin and mineral products and excluding herbal products) using an online questionnaire in Finland, Italy, Poland, and the UK from July 2018 to June 2019. The four countries chosen for the study are geographically and socioeconomically diverse and have different business environments regarding food supplement markets. The target number of participants was 500 from each country, i.e., 2000 participants in total. Pregnant women at any stage of pregnancy able to complete the questionnaire in the main language in each country (i.e., Finnish, Italian, Polish, or English) were included in the study (see Table 1 for participant characteristics). Women who declined to give informed consent were excluded.

Before recruitment, two pilot studies were conducted in each country to test the questionnaires and the feasibility of the recruitment method. In the first pilot study, pregnant or recently delivered women (*n* = 19) completed the questionnaire and gave feedback to the researchers. In the second pilot study, the questionnaire was distributed via social media to women aged 18 to 45 years and feedback was gathered electronically (*n* = 91). After the pilot testing, the questionnaires were refined by clarifying nine questions and providing more response options to four questions.

Recruitment was carried out via a social media platform (Facebook) in each country. A web link to the questionnaire in each language was distributed to the target group of 18- to 45-year-old women. In the UK, the web link was also distributed via other channels, such as childbirth charity research web pages and leisure activity centers.

When accessing the questionnaire, the participants first gave informed consent. If they answered that they were not pregnant or not sure if they were pregnant, they were not able to continue with the questionnaire.

### 2.2. Questionnaire and Supplement Package Information

The research team developed the questions in English and translated them into each language. Subsequently, the questionnaires were translated back to English and necessary modifications were made to ensure the quality and uniformity of the translations. Supplement usage during pregnancy was investigated in detail: data on the type and dose of the supplements used, and the frequency and duration of use were collected.

To calculate the daily intake of each nutrient from the supplements, participants were asked to upload pictures of the supplement packages or web links of the products used. Adherence to supplement recommendations was assessed by comparing each supplement’s daily intake with the supplement recommendations in the relevant country. Folic acid supplementation of 400 µg is recommended for all pregnant women in each of the four countries [16,17,18,19,20], as is vitamin D supplementation in Finland (10 µg) [16,17], Poland (50 µg) [19], and the UK (10 µg) [21]. In Poland, iodine supplementation (200 µg) is also recommended for all pregnant women [19]. Further, supplementation with other micronutrients such as iron, calcium, and magnesium is recommended only for pregnant women at risk of deficient intakes. The daily intakes of supplements were also compared with their safe upper intake levels when available as set by the European Food Safety Authority [22].

Further items included reasons to use food supplements (16 items, response options: yes/no, all reasons listed in Table 2), information sources influencing the decision to use or not to use food supplements (18 items, response options: yes/no, all information sources listed in Table 3), places from which the women bought or obtained food supplements (13 items, response options: yes/no, all places listed in Table S1), and knowledge of relevant dietary recommendations provided by governmental or health bodies on supplement use before and during pregnancy and during breastfeeding (64 items, response options: yes/no, all statements listed in Table S2). Cognitive beliefs (22 items, e.g., awareness of the potential health benefits and risks for the mother and child of supplement use during pregnancy) about supplement use during pregnancy and affective beliefs (2 items) and behavioral intention (2 items) were investigated by asking the participant’s level of agreement with predefined statements (altogether 26 items, response options: strongly agree, agree, disagree, strongly disagree, not sure; full versions of all statements listed in Table 4).

Data to characterize the participants were collected, i.e., stage of pregnancy in weeks, age, European ethnic background, marital status, parity, education, whether working in the health sector, health conditions diagnosed in current pregnancy, non-pregnancy-related health conditions, smoking behavior, alcohol consumption, and dietary habits (i.e., consumption of selected foods typifying the healthiness of a diet [23,24], i.e., frequency of consuming vegetables, fruit and berries, fish, wholegrain, and fast food (response options for each food: consumption 4 times a day or more, 2–3 times a day, once a day, 3–6 times a week, 1–2 times a week, 1–3 times a month, seldom/never)). Pre-pregnancy body mass index (BMI, kg/m^2^) was calculated from the self-reported weight and height and categorized as underweight (BMI < 18.5), normal weight (BMI 18.5–24.9), overweight (BMI ≥ 25.0 and <30.0), and obese (BMI ≥ 30.0) according to guidelines by the World Health Organization [25]. Physical activity during pregnancy was assessed by a validated metabolic equivalent (MET) index that consists of three multiple-choice questions concerning the intensity, frequency, and duration of physical activity [26]. The total MET score ranges from 0 to 105 MET h/week, with scores < 5 indicating light, scores 5–30 moderate, and scores > 30 vigorous physical activity [26].

### 2.3. Statistical Analyses

Continuous data are presented as medians and interquartile ranges, and categorical data as frequencies and percentages. The Kruskal–Wallis test was used for continuous variables and the Chi-square test for categorical variables to analyze between-country differences. Bonferroni corrections were conducted for between-country comparisons. The normality of the data was visually inspected using histograms. The association between food supplement use and sociodemographic (age, pre-pregnancy BMI, marital status, parity, education, whether working in the health sector) and behavioral factors (smoking behavior, alcohol consumption, physical activity, and eating habits) was analyzed using logistic regression analysis, adjusted for country. Each analysis included only one factor at a time (univariate approach with the addition of country). Subsequently, a multivariable model, including significant explanatory variables (country, smoking, and parity), was executed. When assessing the beliefs about and intention to use food supplements, to improve the interpretation and the readability of the tables, ‘strongly agree’ and ‘agree’ and ‘strongly disagree’ and ‘disagree’ answers were combined and participants answering ‘not sure’ were excluded from the analyses. All analyses were 2-tailed, and the level of significance was set to *p*-value < 0.05. Statistical analyses were conducted using SAS version 9.4 for Windows (SAS Institute Inc., Cary, NC, USA) and IBM SPSS Statistics version 27 for Windows (IBM Corp, Armonk, NY, USA).

## 3. Results

### 3.1. Demographic Characteristics

In total, 1804 pregnant women with median pregnancy weeks of 24.7 took part in the study: 536 in Finland, 591 in Italy, 556 in Poland, and 121 in the UK. The demographic characteristics of the participants are presented in Table 1. Significant differences in the characteristics were seen between the countries, e.g., the median weeks of gestation at the time of answering the questionnaire were lowest in Finland and highest in the UK, and the mothers were youngest in Poland and the oldest in the UK and Italy. Most participants were married and primiparous, with both proportions being the lowest in Finland and the highest in Poland. The majority of participants were educated to a university level, with the proportion being highest in the UK and Poland and lowest in Italy. In all countries, almost all participants had a European ethnic background. The consumption of selected foods differed between the countries, namely the proportions of participants eating vegetables at least twice per day, fruit and berries daily, and whole grain products daily were lowest in Italy and highest in Finland while the proportion eating fish weekly was highest in Italy and lowest in Poland. The proportion of participants eating fast food weekly was highest in the UK and lowest in Poland. Participants’ non-pregnancy-related health conditions are presented in Table S3.

### 3.2. Use of Food Supplements

Most participants, 91% (1646/1804), reported using at least one supplement product during pregnancy (98% (526/536) in Finland, 83% (462/556) in Italy, 93% (549/591) in Poland, and 90% (109/121) in the UK). Those using supplements were more likely to be multiparous (*p* = 0.0004) and non-smokers (*p* = 0.047) compared to those not using supplements (Table 5). The results remained essentially the same in the multivariable model (data not shown). Among the food supplement users, the most commonly used (84%, 1387/1646) food supplement types in each country were prenatal multivitamin products, with the proportion of users being lowest in Italy (76%) and highest in Poland (91%) (Table S4). Furthermore, the second most commonly used supplements were iron in Finland (40%), folic acid in Italy (50%), magnesium in Poland (49%), and other multivitamin products (not designed for pregnant women) in the UK (25%).

Food supplements were predominantly used daily or almost daily in all countries, except for in Finland, where iodine, zinc, or vitamin E supplements were typically used less than once a week (Table S4).

**Figure 1:**
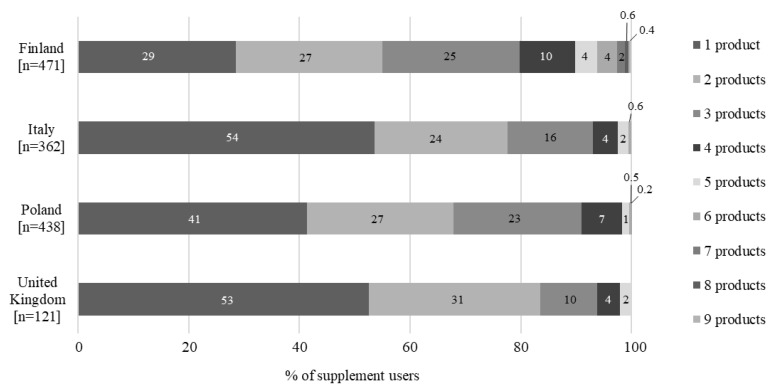
Proportion of supplement users using one or more food supplement products.

The number of supplements used ranged from one to nine products per person; it was most common to use only one product at a time (ranging from 29% of participants in Finland to 54% in Italy, Figure 1). Finnish participants used the highest number of products; one-fifth used more than three food supplements simultaneously, which was a notably higher proportion than in the other countries, where only 5–10% of the participants used more than three products simultaneously.

Supplements were most likely bought from a pharmacy without a prescription in all the countries, and in the UK, supplements were just as likely to be purchased from a supermarket/grocery store (Table S1).

The most common reasons for using food supplements during pregnancy were to support the child’s development during pregnancy (Finland and the UK) and because women had received a recommendation to use the supplements (Poland and Italy, Table 2). Sources of information most likely to influence the women’s decisions to use food supplements were midwives or nurses in Finland and the UK and doctors or general physicians in Italy and Poland. Further, the women’s own intuition was a commonly reported factor in Finland, Poland, and the UK, and the midwife or nurse in Italy (Table 3). Governmental or ministerial web pages were also important information sources in Finland and the UK.

### 3.3. Adherence to Food Supplement Recommendations

The total intakes of nutrients from the food supplements as calculated from the supplement package labels and adherence to food supplement recommendations and the current food supplement recommendations in each country studied are reported in Table 6. Most participants in all countries used folic acid supplements; the proportion ranged from 93% in Finland to 98% in Poland. In the UK, 90% of the folic acid users used the recommended dose while only 26% adhered to the recommendation in Poland. In Finland, 11% (46/437) of folic acid users used less than the recommended dose while the respective proportions were notably lower in the other countries: 3% (10/342) in Italy, 4% (17/432) in Poland, and 2% (2/92) in the UK.

In Finland, Poland, and the UK, it is recommended to use vitamin D as a food supplement during pregnancy and 97%, 91%, and 95% of participants, respectively, reported complying with this (Table 6). However, in Finland and Poland, less than 60% of these women used the recommended dose of vitamin D. In the UK, the adherence to vitamin D recommendations was higher as 84% of the participants used the recommended dose. In Poland, 36% (144/399) of vitamin D users used less than the recommended amount of vitamin D, whereas the respective proportion was 9% (39/456) in Finland and 2% (2/92) in the UK. In Italy, there is no recommendation for the use of vitamin D during pregnancy; nevertheless, 77% of the participants used vitamin D as a supplement.

In Poland, 86% of the participants complied with the recommendation of iodine supplementation (Table 6). Almost two-thirds of the iodine supplement users adhered to the recommended intakes for iodine supplements and one-third (123/378) obtained less iodine compared to the recommendations.

Exceeding the recommended food supplement doses was relatively common, especially in Poland, where over two-thirds of folic acid supplement users exceeded the recommended doses for these nutrients (Table 6). By comparison, only 8% of folic acid users in the UK exceeded the recommended dose. In 19% (265/1364) of the supplement users reporting their daily nutrient intakes from food supplements, the daily safe upper intake limit of at least one nutrient was exceeded, with the proportions being 20% (92/470) of the supplement users in Finland, 25% (91/360) in Italy, 17% (76/437) in Poland, and 6% (6/97) in the UK. The proportions of participants exceeding the daily safe upper intake limits were the highest with magnesium supplements in each country (Table 6). The daily intakes of nutrients from the food supplements with no recommendations during pregnancy are reported in Table S5.

### 3.4. Awareness Regarding Food Supplements during Pregnancy

**Figure 2:**
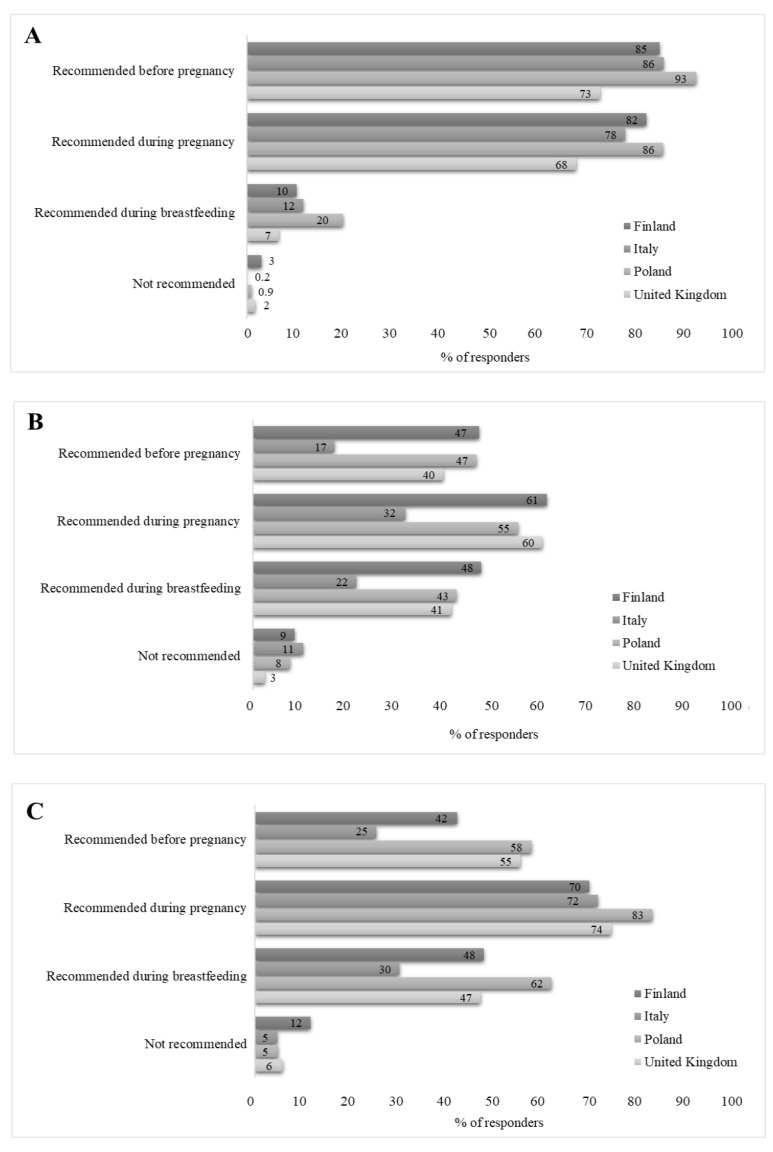
Proportion of participants reporting in an online survey that specific supplements are recommended for use before and during pregnancy and during breastfeeding by country of residence: (**A**) folic acid; (**B**) vitamin D; (**C**) multivitamin supplement. Finland, *n* = 536; Italy, *n* = 540; Poland, *n* = 585; the United Kingdom, *n* = 121.

Most participants agreed with the statements that they know which food supplements (91%, 1520/1676) and what doses of supplements (87%, 1437/1661) they need to consume during pregnancy, with both proportions being lowest in the UK and highest in Finland (Table 4). The majority of the participants (81%, 1456/1795) knew that folic acid is recommended during the first months of pregnancy; the recommendation was most familiar in Poland and least familiar in the UK (Figure 2). In Finland, Poland, and the UK, less than two-thirds of the participants knew that vitamin D supplementation is recommended during pregnancy in their home country while one-third of the Italian participants mistakenly thought vitamin D supplementation is recommended during pregnancy in Italy. Moreover, 75% (1346/1795) thought that multivitamin supplements are generally recommended during pregnancy, with the proportion being the lowest in Finland and the highest in Poland. In general, there was wide variation in the knowledge of which nutrients are recommended as food supplements during pregnancy between countries (Table S2).

## 4. Discussion

The results of this multinational survey indicate that the consumption of food supplements during pregnancy is common in the four countries of the study. Regardless, the recommendations for food supplement use were not universally known and the calculation of the nutrient intakes from the supplement packages revealed that the daily safe upper intake limit of at least one nutrient was exceeded in one-fifth of the food supplement users. Country differences were detected in the supplement use, adherence to the recommendations, and awareness of food supplement recommendations and beliefs about food supplement use during pregnancy.

To our knowledge, this is the first multinational survey to assess the daily intakes of nutrients from food supplements during pregnancy. The results indicate that it is relatively common to use higher than the recommended doses of supplements and even exceed the daily safe upper nutrient intake levels. Previous multinational studies on food supplement use during pregnancy [9,10] have mainly reported the percentages of women using vitamins and minerals as food supplements rather than the specific amounts of supplements taken. Our results raise concerns over excessive use of some supplements due to potential adverse effects on the health of the mother and fetus, e.g., teratogenicity of vitamin A. Although the participants did not use vitamin A supplements above the daily safe upper intake limit, in Italy, almost every tenth participant used a supplement containing vitamin A, which is not recommended to be used at all as a supplement during pregnancy. Further, the daily safe upper intake limit of magnesium was commonly exceeded and, although severe adverse effects are seen with 10-fold doses compared to the safe upper intake limit [22], it does seem that a large proportion of pregnant women are not aware of the recommendations and safe doses.

The result that the food supplement use deviated from the recommendations is in line with the finding that the mothers’ knowledge on the supplement recommendations also varied. The recommendations for the use of folic acid before and during pregnancy were known by four out of five participants, likely reflecting the fact that the adverse consequences of low intake of folic acid on the normal development of a child’s brain and nervous system are well known. Interestingly, previous studies conducted in Italy, Ireland, and Portugal have noted that, even in the case of planned pregnancies, only some women used folic acid before conception and during the early stages of the pregnancy despite the recommendations and available public health programs [27,28,29]. It is of note that although folic acid is needed to prevent neural tube defects mainly in preconception and early pregnancy, it has a crucial role in the development of the fetus in many other ways throughout pregnancy, e.g., in hematopoiesis, nucleic acid synthesis, and gene regulation. In addition to folic acid, vitamin D is recommended to be used as a supplement in the studied countries, bar Italy. Nevertheless, only about half of the participants were aware of the recommendation for vitamin D in Finland, the UK, and Poland; and one-third of the Italian participants thought vitamin D use is recommended during pregnancy. In the case of multivitamins, where there are no recommendations for all pregnant women to use them, three out of four participants thought that multivitamins were recommended. The question on the sources of information indicated that, while health care professionals were the main sources of information, many women also rely on their own initiative, other pregnant women, and social media, which might sometimes lead to inaccurate presumptions and thus explain these findings.

All in all, the majority of pregnant women used at least one food supplement, with prenatal multivitamin products being the most common. Similar findings have been reported in previous studies among pregnant women in Australia, the USA, and Canada [30,31,32,33,34]. In our study, most participants used one or two supplements simultaneously, however, interestingly, in Finland, up to nine products were used. A previous survey found that the proportion of non-pregnant consumers using two or more plant food supplements simultaneously was significantly higher in Finland than in all other countries [35]. The reasons for Finnish women consuming multiple food supplements remain unknown but may relate to differences in advertising or healthcare.

We sought to characterize food supplement users and found that primiparous and non-smoking women were more likely to consume food supplements as compared to multiparous and smoking women, with this result being supported by findings from previous studies with both pregnant [9,34,36,37] and non-pregnant women [38,39,40]. Surprisingly, we did not detect an association between supplement use and some factors previously linked with their use during pregnancy such as education, maternal age, or BMI [9,12,13,37,41,42,43,44,45]. It could be speculated whether supplement use is linked to a general awareness, irrespective of these factors, of the importance of sufficient nutrient intake during pregnancy for the benefit of the mother and child. Indeed, in our study, one of the most common reasons for supplement use was that women wanted to support their child’s development. Additionally, other studies have reported the reasons for supplement use, including recommendation from a health care provider [41,46] and increased nutrient requirements due to pregnancy [43]. However, as demonstrated in our study and also in those of others, the reasons for supplement use [41,43,46], information sources [32,42,47] and places of supplement purchase [42,48] differ between countries, which may be explained by differences in the health care systems, media coverage, and cultural differences with regard to pregnancy. In Poland and Italy, pregnant women primarily received pregnancy-related advice from obstetricians whereas in Finland and the UK, women received advice from nurses or midwives.

This study has several strengths, including the detailed and unique data on daily supplement intake calculated from the packages, the large sample size, and the recruitment in four geographically and socioeconomically diverse European countries, which enabled the investigation of country differences. Appropriate measures were also taken in preparing the study questionnaires, including pilot studies and translation procedures. Participants were recruited via social media, which is nowadays considered a modern and, importantly, an effective recruitment method in observational studies [49] and is especially suitable for recruiting younger females and hard-to-reach populations [50]. Recruitment via social media may also be a potential source of bias as the participants might gain supplement-related information. In this study, it is likely not to be relevant as only a few women reported social media being an important source of supplement information (Table 3).

This study also has limitations. Firstly, regardless of the successful pilot study and use of additional recruitment methods, the recruitment in the UK was not as effective as in the other countries. Nonetheless, the UK data was considered valuable to be reported, but the smaller sample size needs to be considered as a limitation. Secondly, women who were recruited were using and/or were interested in food supplements, thus possibly impacting the results, including that the non-user group was rather small. However, similar proportions of supplement users have been found in previous studies, which were mainly part of cohort or clinical studies [8,9,31,32,36,37]. Thirdly, social desirability bias may have potentially impacted the answers, e.g., if participants overestimate the use of recommended supplements and underestimate that of other supplements. However, we anticipate that the risk of bias is low as the questionnaire was anonymous and completed electronically. In addition, the self-reporting of the data should be considered as a potential source of error while interpreting the results. However, the calculation of the daily supplement intakes from the package labels diminishes the potential inaccuracies that may be relevant when inquiring about supplement use using a questionnaire. It is of note that the majority of the participants (76%, 1366/1804) provided information and pictures of the supplement packages or web links of the products used. Furthermore, it is possible that the consumption of high doses of some nutrients by some of the women might have been because they were prescribed high doses of folic acid due to a risk for neural tube defects, or high doses of iron in the case of anemia. Lastly, the proportion of women with a university-level education was higher than the national average in adults (19–36%) in each country [51]. Since people with high education levels might be more likely to consume food supplements [9,12,37,43,44] and have a better knowledge of supplement recommendations [52,53] compared to those with a low education level, the results might not be fully applicable to women with a lower education level, although we did not observe an association between education level and supplement use. Further, while interpreting the results of the country differences (Table 2, Table 3, Table 4 and Table 6), it needs to be considered that differences in the demographic variables between the countries could contribute to the findings. However, the country variable in the analyses represents both the country effect and the demographic effect due to collinearity. Despite these limitations, our study provides novel and useful information about supplement use during pregnancy.

## 5. Conclusions

Although a majority of the participants used food supplements and felt that they knew which supplements and what doses of supplements they should use during pregnancy, in practice, the number of participants consuming relevant supplements and, especially, those adhering to supplement recommendations was lower. There was also wide variation in the use and knowledge of and beliefs regarding supplements between countries. The results suggest a need for enhanced assessment of food supplement use and initiatives to ensure the optimal use of supplements during pregnancy. Greater alignment of food supplement recommendations across countries would allow for more globally integrated public health messaging, thus reducing confusion regarding recommendations.

## Figures and Tables

**Table 1:** Characteristics of the pregnant women participating in the study (*n* = 1804).

		All	Finland	Italy	Poland	United Kingdom	*p*-Value
	Total *n*	*n* (%) or Median (IQR)					
**Pregnancy weeks**	1803/536/555/591/121	24.7 (16.7–32.4)	24.4 (15.2–31.7)	24.4 (16.7–31.3)	25.4 (17.1–33.3)	25.1 (18.0–33.4)	<0.001 ^a^
**Age, years**	1794/535/551/587/121	30.0 (27.0–33.0)	30.0 (27.0–33.0)	32.0 (29.0–35.0)	28.0 (25.0–31.0)	32.0 (29.0–35.0)	<0.001 ^a^
<25 years		189 (10.5)	45 (8.4)	22 (4.0)	106 (18.1)	16 (13.2)	<0.001 ^b^
25–29 years		662 (36.9)	217 (40.6)	142 (25.8)	283 (48.2)	20 (16.5)	
30–34 years		633 (35.3)	201 (37.6)	222 (40.3)	162 (27.6)	48 (39.7)	
≥35 years		310 (17.3)	72 (13.5)	165 (30.0)	36 (6.1)	37 (30.6)	
**Pre-pregnancy BMI**	1774/534/547/586/107	23.1 (20.7–26.3)	24.1 (21.5–27.6)	22.6 (20.4–25.5)	22.7 (20.5–25.5)	23.4 (21.5–27.1)	<0.001 ^a^
Underweight		102 (5.7)	11 (2.1)	40 (7.3)	48 (8.2)	3 (2.8)	<0.001 ^b^
Normal weight		1111 (62.6)	313 (58.6)	365 (66.7)	369 (63.0)	64 (59.8)	
Overweight		362 (20.4)	112 (21.0)	93 (17.0)	133 (22.7)	24 (22.4)	
Obese		199 (11.2)	98 (18.4)	49 (9.0)	36 (6.1)	16 (15.0)	
**European ethnic background**	1788/535/547/587/119	1773 (99.2)	535 (100.0)	539 (98.5)	587 (100.0)	112 (94.1)	<0.001 ^b^
**Marital status**	1793/534/551/588/120						<0.001 ^b^
Married		1131 (63.1)	286 (53.6)	320 (58.1)	442 (75.2)	83 (69.2)	
Living with a partner		614 (34.2)	227 (42.5)	225 (40.8)	132 (22.4)	30 (25.0)	
Other		48 (2.7)	21 (3.9)	6 (1.1)	14 (2.4)	7 (5.8)	
**Parity**	1795/535/551/588/121						<0.001 ^b^
1st child		1205 (67.1)	336 (62.8)	359 (65.2)	430 (73.1)	80 (66.1)	
2nd child		458 (25.5)	132 (24.7)	168 (30.5)	124 (21.1)	34 (28.1)	
3rd child or more		132 (7.4)	67 (12.5)	24 (4.4)	34 (5.8)	7 (5.8)	
**Education**	1796/536/551/588/121						<0.001 ^b^
Secondary education or less		550 (30.6)	157 (29.3)	235 (42.7)	131 (22.3)	27 (22.3)	
University		1246 (69.4)	379 (70.7)	316 (57.4)	457 (77.7)	94 (77.7)	
**Working in the health sector**	1712/502/515/576/119	476 (27.8)	229 (45.6)	119 (23.1)	88 (15.3)	40 (33.6)	<0.001 ^b^
**Smoking**							
Before pregnancy	1792/534/551/585/121	559 (31.2)	108 (20.2)	248 (45.0)	183 (31.2)	20 (16.5)	<0.001 ^b^
During pregnancy	1796/534/556/585/121	97 (5.4)	14 (2.6)	49 (8.9)	32 (5.5)	2 (1.7)	<0.001 ^b^
**Alcohol consumption**							
Before pregnancy	1792/535/549/587/121	1431 (79.9)	448 (83.7)	393 (71.6)	482 (82.1)	108 (89.3)	<0.001 ^b^
During pregnancy	1791/535/549/587/120	99 (5.5)	8 (1.5)	71 (13.0)	5 (0.9)	15 (12.5)	<0.001 ^b^
**Physical activity level during** **pregnancy**	1774/531/541/581/121						<0.001 ^b^
Low activity		1259 (71.0)	268 (50.5)	448 (82.8)	480 (82.6)	63 (52.1)	
Moderate activity		467 (26.3)	224 (42.2)	89 (16.5)	98 (16.9)	56 (46.3)	
High activity		48 (2.7)	39 (7.3)	4 (0.7)	3 (0.5)	1 (1.7)	
**Dietary habits during pregnancy**							
Vegetables ≥ 2 times/day	1795/536/550/588/121	777 (43.3)	315 (58.8)	113 (20.5)	281 (47.8)	68 (56.2)	<0.001 ^b^
Fruits and/or berries daily	1791/534/550/587/120	1252 (69.9)	399 (74.7)	334 (60.7)	430 (73.3)	89 (74.2)	<0.001 ^b^
Whole grain products daily	1793/535/551/587/120	979 (54.6)	420 (78.5)	183 (33.2)	299 (50.9)	77 (64.2)	<0.001 ^b^
Fish weekly	1792/535/550/587/120	954 (53.2)	276 (51.6)	372 (67.6)	242 (41.2)	64 (53.3)	<0.001 ^b^
Fast food weekly	1794/536/551/586/121	388 (21.7)	163 (30.4)	117 (21.2)	63 (10.8)	45 (37.2)	<0.001 ^b^
**Health conditions diagnosed in** **current pregnancy**	1791/536/549/585/121						
Gestational diabetes		143 (8.0)	62 (11.6)	26 (4.7)	52 (8.9)	3 (2.5)	<0.001 ^b^
Pre-eclampsia/high blood pressure		30 (1.7)	7 (1.3)	10 (1.8)	13 (2.2)	0 (0.0)	1.000 ^b^
Pregnancy related nausea		632 (35.3)	213 (39.7)	208 (37.9)	159 (27.2)	52 (43.0)	<0.001 ^b^

^a^ Comparison between the countries, Kruskal–Wallis test with Bonferroni correction. ^b^ Comparison between the countries, Chi-square test with Bonferroni correction.

**Table 2:** Reasons for using food supplements during pregnancy reported by pregnant women in four European countries (proportion of women answering ‘yes’ to the statements).

	Finland	Italy	Poland	United Kingdom	*p*-Value ^b^
**Statement, *n* (%) ^a^**	*n* = 536	*n* = 468	*n* = 553	*n* = 111	
I consume food supplements…					
as consuming supplements has been recommended for me	407 (75.9)	402 (85.9)	398 (72.0)	78 (70.3)	<0.001
as I think I don’t get enough nutrients from food	237 (44.2)	43 (9.2)	260 (47.0)	26 (23.4)	<0.001
as I think consuming supplements is beneficial for me	376 (70.2)	91 (19.4)	257 (46.5)	42 (37.8)	<0.001
on a regular basis also when not pregnant	160 (29.9)	39 (8.3)	45 (8.1)	18 (16.2)	<0.001
to be on the safe side	94 (17.5)	31 (6.6)	59 (10.7)	36 (32.4)	<0.001
to improve my vitality	95 (17.7)	38 (8.1)	47 (8.5)	9 (8.1)	<0.001
to sleep better	38 (7.1)	4 (0.9)	5 (0.9)	3 (2.7)	<0.001
to enhance my gut well-being	94 (17.5)	25 (5.3)	20 (3.6)	9 (8.1)	<0.001
to enhance my immune system	141 (26.3)	31 (6.6)	53 (9.6)	14 (12.6)	<0.001
to manage my disease	14 (2.6)	17 (3.6)	34 (6.2)	2 (1.8)	0.078
to be healthy in general	148 (27.6)	76 (16.2)	117 (21.2)	14 (12.6)	<0.001
to support exercise	31 (5.8)	0 (0.0)	5 (0.9)	4 (3.6)	<0.001
as I think consuming food supplements is beneficial for my baby	422 (78.7)	199 (42.5)	369 (66.7)	76 (68.5)	<0.001
to support the development of my baby	428 (79.9)	238 (50.9)	396 (71.6)	82 (73.9)	<0.001
to support the growth of my baby	313 (58.4)	214 (45.7)	284 (51.4)	71 (64.0)	<0.001
Other reason ^c^	34 (6.3)	19 (4.1)	22 (4.0)	6 (5.4)	1.000

^a^ Proportion of participants answering ‘yes’. ^b^ Comparison between the countries, Chi-square test with Bonferroni correction. ^c^ Health reasons such as anemia, cramps, restless legs, nausea, vaginal health, and previous gastric bypass; deficiency of some vitamin or mineral; dietary reasons such as following a vegan or vegetarian diet; breastfeeding.

**Table 3:** Information sources reported by pregnant women in four European countries that influence their food supplements use.

	Finland	Italy	Poland	United Kingdom	*p*-Value ^b^
**Information source, *n* (%) ^a^**	*n* = 536	*n* = 540	*n* = 585	*n* = 121	
Partner	59 (11.0)	19 (3.5)	39 (6.7)	15 (12.4)	<0.001
Parents or other relatives	46 (8.6)	14 (2.6)	34 (5.8)	13 (10.7)	<0.001
Friends	87 (16.2)	5 (0.9)	30 (5.1)	13 (10.7)	<0.001
Own initiative	268 (50.0)	126 (23.3)	261 (44.6)	84 (69.4)	<0.001
Other pregnant women or mothers	115 (21.5)	57 (10.6)	147 (25.1)	19 (15.7)	<0.001
Midwife/nurse	431 (80.4)	168 (31.1)	75 (12.8)	90 (74.4)	<0.001
Nutritionist/dietitian	28 (5.2)	27 (5.0)	12 (2.1)	9 (7.4)	<0.001
Doctor/general physician	135 (25.2)	361 (66.9)	446 (76.2)	34 (28.1)	<0.001
Pharmacy personnel	103 (19.2)	26 (4.8)	43 (7.4)	0 (0.0)	<0.001
Herbal shop personnel	18 (3.4)	5 (0.9)	0 (0.0)	0 (0.0)	<0.001
Adverts	13 (2.4)	1 (0.2)	15 (2.6)	9 (7.4)	<0.001
Books	31 (5.8)	4 (0.7)	35 (6.0)	12 (9.9)	<0.001
Magazines or newspapers	29 (5.4)	4 (0.7)	20 (3.4)	5 (4.1)	<0.001
Social media	85 (15.9)	8 (1.5)	53 (9.1)	16 (13.2)	<0.001
Mobile applications (apps)	14 (2.6)	11 (2.0)	8 (1.4)	5 (4.1)	1.000
Governmental or ministerial websites	197 (36.8)	11 (2.0)	10 (1.7)	30 (24.8)	<0.001
Commercial websites, blogs	65 (12.1)	5 (0.9)	66 (11.3)	4 (3.3)	<0.001
Other ^c^	39 (7.3)	6 (1.1)	16 (2.7)	7 (5.8)	<0.001

^a^ Proportion of participants answering ‘yes’. ^b^ Comparison between the countries, Chi-square test with Bonferroni correction. ^c^ TV or radio, own education or work experience, scientific literature, online pharmacy.

**Table 4:** Proportion of women agreeing with the statements regarding beliefs about and intention to use food supplements during pregnancy.

	Total *n*	Finland	Italy	Poland	United Kingdom	*p*-Value ^a,b^
**Statement**		***n* (%) of women agreeing with the statement**				
**Cognitive statements**						
I know which food supplements I need to consume during pregnancy	523/515/526/112	497 (95.0)	447 (86.8)	483 (91.8)	93 (83.0)	<0.001
I know what doses of food supplements I need to consume during pregnancy	527/502/527/105	482 (91.5)	425 (84.7)	460 (87.3)	70 (66.7)	<0.001
I understand how food supplements may impact my health	517/492/537/106	480 (92.8)	393 (79.9)	490 (91.2)	75 (70.8)	<0.001
I believe that consuming food supplements during pregnancy benefit my health, as an expectant mother	522/513/549/104	514 (98.5)	458 (89.3)	524 (95.4)	93 (89.4)	<0.001
I’m unsure of how food supplements benefit the health of my baby	506/506/545/113	88 (17.4)	170 (33.6)	115 (21.1)	38 (33.6)	<0.001
It is easy for me to regularly consume food supplements	533/518/570/116	463 (86.9)	410 (79.2)	461 (80.9)	90 (77.6)	0.024
More nutrients are needed during pregnancy than before pregnancy	509/522/583/107	489 (96.1)	474 (90.8)	574 (98.5)	99 (92.5)	<0.001
Absorption of nutrients from the gut is more efficient during pregnancy than when not pregnant	216/324/181/32	156 (72.2)	112 (34.6)	53 (29.3)	15 (46.9)	<0.001
A diverse and healthy diet is the best way to get most nutrients I need	520/520/550/112	481 (92.5)	478 (91.9)	529 (96.2)	108 (96.4)	0.060
It is necessary to consume particular food supplements in addition to dietary intake during pregnancy	471/481/527/98	248 (52.7)	389 (80.9)	464 (88.0)	84 (85.7)	<0.001
Food supplements used help the baby to grow well	451/479/520/104	366 (81.2)	420 (87.7)	478 (91.9)	89 (85.6)	<0.001
Food supplements used help the baby to develop well	496/485/536/109	479 (96.6)	449 (92.6)	508 (94.8)	103 (94.5)	0.306
Food supplements used help the baby to be healthy	393/464/512/105	333 (84.7)	376 (81.0)	478 (93.4)	92 (87.6)	<0.001
Food supplements are medicines	502/481/526/98	53 (10.6)	98 (20.4)	69 (13.1)	32 (32.7)	<0.001
Vitamin A is important for the baby’s vision and eye development	265/260/322/62	144 (54.3)	224 (86.2)	249 (77.3)	34 (54.8)	<0.001
A very high intake of vitamin A from supplements during pregnancy may be harmful for the baby	444/239/343/93	441 (99.3)	162 (67.8)	322 (93.9)	92 (98.9)	<0.001
Vitamin D is needed to ensure the absorption of calcium during pregnancy	458/337/379/90	453 (98.9)	318 (94.4)	364 (96.0)	88 (97.8)	0.018
Pregnant women generally get enough vitamin D from their food in my country	481/297/408/104	35 (7.3)	95 (32.0)	31 (7.6)	13 (12.5)	<0.001
Pregnant women generally get enough folate from their food in my country	489/362/466/97	25 (5.1)	61 (16.9)	64 (13.7)	10 (10.3)	<0.001
Insufficient intake of folic acid may disturb the development of brain and nervous system of the baby	498/499/552/112	484 (97.2)	475 (95.2)	544 (98.6)	108 (96.4)	0.096
Sufficient intake of iron during pregnancy prevents tiredness of the mother	507/472/356/99	487 (96.1)	424 (89.8)	329 (92.4)	84 (84.8)	<0.001
Iodine is essential for the baby’s brain and nerve development	193/221/326/32	181 (93.8)	196 (88.7)	312 (95.7)	30 (93.8)	0.096
**Affective statements**						
I’m afraid that consuming food supplements during pregnancy may be harmful for my baby	501/511/537/114	35 (7.0)	35 (6.8)	43 (8.0)	13 (11.4)	1.000
I’m troubled by the thought that food supplements are highly processed	471/481/472/109	119 (25.3)	103 (21.4)	147 (31.1)	21 (19.3)	0.120
**Behavioral statements**						
I’m more willing to consume food supplements when I’m not pregnant than when I am pregnant	478/490/546/117	72 (15.1)	86 (17.6)	96 (17.6)	22 (18.8)	1.000
I intend to consume food supplements throughout my pregnancy	520/499/557/114	499 (96.0)	431 (86.4)	514 (92.3)	98 (86.0)	<0.001

^a^ Comparison of the proportion of women agreeing and disagreeing with the statements between the countries. ‘Not sure’ answers were removed from the analyses. ^b^ Comparison between the countries, Chi-square test with Bonferroni correction.

**Table 5:** Likelihood of any food supplement use according to selected sociodemographic and behavioral factors characterizing pregnant women in four European countries.

	Total *n*	Adjusted OR ^a^	95 % Confidence Interval	*p*-Value ^b^
**Sociodemographic factors**				
**Age, years**	1794			0.48
<25 years		1		
25–29 years		1.513	0.828–2.766	
30–34 years		1.255	0.690–2.281	
≥35 years		1.128	0.590–2.157	
**Pre-pregnancy BMI**	1774			0.50
Underweight		1		
Normal weight		1.476	0.796–2.736	
Overweight		1.225	0.617–2.434	
Obese		1.689	0.730–3.905	
**Marital status**	1793			0.063
Living with a partner		1		
Married		1.343	0.943–1.911	
Other		0.546	0.213–1.399	
**Parity**	1795			0.0004
1st child		1		
2nd or more		1.852	1.317–2.606	
**Education**	1796			0.47
Secondary education or less		1		
University		1.140	0.800–1.625	
**Working in the health sector**	1712			0.52
No		1		
Yes		1.153	0.751–1.770	
**Behavioral factors**				
**Regular smoking before pregnancy**	1794			0.047
No		1		
Yes		0.676	0.460–0.994	
**Alcohol consumption before pregnancy**	1792			0.45
Not at all		1		
<1 drink per week		1.396	0.909–2.141	
1–2 drinks per week		1.159	0.731–1.837	
3–7 drinks per week		1.740	0.837–3.615	
>7 drinks per week		0.940	0.259–3.409	
**Physical activity level during pregnancy**	1774			0.73
Low activity		1		
Moderate activity		0.894	0.586–1.364	
High activity		1.796	0.233–13.811	
**Consuming vegetables ≥ 2 times/day**	1795			0.47
No		1		
Yes		1.151	0.787–1.682	
**Consuming fruits and/or berries daily**	1791			0.75
No		1		
Yes		0.942	0.658–1.350	
**Consuming whole grain products daily**	1635			0.83
No		1		
Yes		1.038	0.730–1.475	
**Consuming fish weekly**	1792			0.91
No		1		
Yes		0.980	0.690–1.391	

^a^ Adjusted by country. ^b^ Logistic regression analysis with a univariate approach.

**Table 6:** Daily intakes of nutrients from food supplements and adherence to food supplement recommendations in pregnant women who reported their supplement use with pictures and/or detailed written information.

	Finland	Italy	Poland	United Kingdom	*p*-Value
**Nutrient**	*n* = 470	*n* = 360	*n* = 439	*n* = 97	
**Vitamin A, µg/d**					
User, *n* (%)	4 (0.9)	31 (8.6)	7 (1.6)	1 (1.0)	<0.001 ^a^
Dose, median (IQR)	400.0 (325.0–700.0)	300.0 (300.0–300.0)	800.0 (640.0–800.0)	800.0	0.036 ^b^
Dose, range	300.0–800.0	300.0–1080.0	400,0–1080.0	-	
Women exceeding the daily safe upper intake limit of 3000 µg/d ^c^, *n* (%)	0 (0.0)	0 (0.0)	0 (0.0)	0 (0.0)	
**Vitamin B6 (pyridoxine), mg/d**					
User, *n* (%)	404 (86.0)	284 (78.9)	310 (70.6)	79 (81.4)	<0.001 ^a^
Dose, median (IQR)	5.0 (2.8–5.0)	1.9 (1.4–1.9)	2.6 (2.2–5.0)	10.0 (1.9–10.0)	<0.001 ^b^
Dose, range	0.5–51.2	0.6–20.9	0.6–100.0	1.4–30.0	
Women exceeding the daily safe upper intake limit of 25 mg/d ^c^, *n* (%)	6 (1.5)	0 (0.0)	16 (5.2)	1 (1.3)	
**Vitamin B9 (folic acid), µg/d ^d^**					
User, *n* (%)	437 (93.0)	342 (95.0)	432 (98.4)	92 (94.8)	0.006 ^a^
Dose, median (IQR)	400.0 (400.0–500.0)	400.0 (400.0–400.0)	800.0 (400.0–800.0)	400.0 (400.0–400.0)	<0.001 ^b^
Dose, range	100.0–1500.0	142.9–16200.0	114.3–6600.0	171.4–5400.0	
Women meeting the recommended dose, *n* (%)	240 (54.9)	258 (75.4)	112 (26.0)	83 (90.2)	
Women exceeding the recommended dose, *n* (%)	151 (34.6)	74 (21.6)	303 (70.1)	7 (7.6)	
Women exceeding the daily safe upper intake limit of 1000 µg/d ^c^, *n* (%)	7 (1.6)	43 (12.6)	43 (10.0)	2 (2.2)	
**Vitamin D, µg/d ^e^**					
User, *n* (%)	456 (97.0)	277 (76.9)	399 (90.9)	92 (94.8)	<0.001 ^a^
Dose, median (IQR)	10.0 (10.0–20.0)	12.5 (10.0–15.0)	50.0 (20.0–50.0)	10.0 (10.0–10.0)	<0.001 ^b^
Dose, range	0.9–253.3	3.6–250.0	2.5–332.5	4.3–85.0	
Women meeting the recommended dose, *n* (%)	266 (58.3)	-	224 (56.1)	77 (83.7)	
Women exceeding the recommended dose, *n* (%)	151 (33.1)	-	31 (7.8)	13 (14.1)	
Women exceeding the daily safe upper intake limit of 100 µg/d ^c^, *n* (%)	9 (2.0)	5 (1.8)	7 (1.8)	0 (0.0)	
**Vitamin E, mg/d**					
User, *n* (%)	385 (81.9)	166 (46.1)	215 (49.0)	71 (73.2)	<0.001 ^a^
Dose, median (IQR)	15.0 (12.0–15.0)	12.0 (8.0–12.0)	11.7 (11.7–23.4)	4.0 (4.0–12.0)	<0.001 ^b^
Dose, range	2.3–30.0	3.0–280.0	1.0–130.0	1.7–200.0	
Women exceeding the daily safe upper intake limit of 300 mg/d ^c^, *n* (%)	0 (0.0)	0 (0.0)	0 (0.0)	0 (0.0)	
**Calcium, mg/d**					
User, *n* (%)	113 (24.0)	114 (31.7)	40 (9.1)	36 (37.1)	<0.001 ^a^
Dose, median (IQR)	500.0 (400.0–750.0)	140.0 (140.0–242.5)	200.0 (200.0–240.0)	200.0 (120.0–500.0)	<0.001 ^b^
Dose, range	42.9–1100.0	36.9–731.0	70.0–1000.0	120.0–1120.0	
Women exceeding the daily safe upper intake limit of 2500 mg/d ^c^, *n* (%)	0 (0.0)	0 (0.0)	0 (0.0)	0 (0.0)	
**Magnesium, mg/d**					
User, *n* (%)	390 (83.0)	181 (50.3)	241 (54.9)	77 (79.4)	<0.001 ^a^
Dose, median (IQR)	180.0 (180.0–180.0)	110.0 (60.0–110.0)	90.0 (50.0–193.0)	150.0 (150.0–150.0)	<0.001 ^b^
Dose, range	37.5–930.0	9.0–1140.0	2.4–600.0	60.0–410.0	
Women exceeding the daily safe upper intake limit of 250 mg/d ^c^, *n* (%)	76 (19.5)	52 (28.7)	28 (11.6)	3 (3.9)	
**Zinc, mg/d**					
User, *n* (%)	382 (81.3)	266 (73.9)	99 (22.6)	77 (79.4)	<0.001 ^a^
Dose, median (IQR)	15.0 (10.7–15.0)	10.0 (10.0–11.0)	15.0 (11.0–15.0)	15.0 (15.0–15.0)	<0.001 ^b^
Dose, range	2.0–40.0	0.9–22.5	3.8–101.0	6.4–25.0	
Women exceeding the daily safe upper intake limit of 25 mg/d ^c^, *n* (%)	4 (1.0)	0 (0.0)	2 (2.0)	0 (0.0)	
**Selenium, µg/d**					
User, *n* (%)	369 (78.5)	210 (58.3)	138 (31.4)	77 (79.4)	<0.001 ^a^
Dose, median (IQR)	60.0 (55.0–60.0)	55.0 (30.0–55.0)	55.0 (55.0–55.0)	30.0 (30.0–55.0)	<0.001 ^b^
Dose, range	12.5–88.0	12.5–112.5	16.0–200.0	12.9–150.0	
Women exceeding the daily safe upper intake limit of 300 µg/d ^c^, *n* (%)	0 (0.0)	0 (0.0)	0 (0.0)	0 (0.0)	
**Iodine, µg/d ^f^**					
User, *n* (%)	379 (80.6)	266 (3.9)	378 (86.1)	76 (78.4)	<0.001 ^a^
Dose, median (IQR)	175.0 (175.0–200.0)	200.0 (175.0–220.0)	200.0 (150.0–200.0)	150.0 (150.0–150.0)	<0.001 ^b^
Dose, range	37.5–220.0	64.3–440.0	50.0–400.0	50.0–290.0	
Women meeting the recommended dose, *n* (%)	-	-	241 (63.8)	-	
Women exceeding the recommended dose, *n* (%)	-	-	14 (3.7)	-	
Women exceeding the daily safe upper intake limit of 600 µg/d ^c^, *n* (%)	0 (0.0)	0 (0.0)	0 (0.0)	0 (0.0)	

^a^ Comparison between the countries, Chi-square test with Bonferroni correction. ^b^ Comparison between the countries, Kruskal–Wallis test with Bonferroni correction. ^c^ Safe upper intake limit reported as set by the European Food Safety Authority [22]. ^d^ Recommended intake of folic acid from supplements during pregnancy: 400 µg/d in each country in the low-risk group. ^e^ Recommended intake of vitamin D from supplements during pregnancy: 10 µg/d in Finland and the UK, 50 µg/d in Poland, no recommendation in Italy. ^f^ Recommended intake of iodine from supplements during pregnancy: 200 µg/d in Poland, no recommendation in other countries.

## Data Availability

The data presented in this study are available upon reasonable request from the last author (K.L.). The data are not publicly available as they contain information that could compromise the privacy of the research participants.

## Supplementary Materials

The following supporting information can be downloaded at: https://www.mdpi.com/article/10.3390/nu14142909/s1, Table S1: Places from which the women bought or obtained food supplements reported by pregnant women in four European countries; Table S2: Perceived recommendations for food supplement use before and during pregnancy and during breastfeeding as reported by pregnant women in four European countries; Table S3: Non-pregnancy-related health conditions of the pregnant women participating in the study; Table S4: Frequency of food supplement use during pregnancy as reported by pregnant women in four European countries; Table S5: Daily intakes of nutrients from food supplements with no recommended intake levels during pregnancy.
