# Supplementary material for: Food Supplement Use Differs from the Recommendations in Pregnant Women: A Multinational Survey
Source: Nutrients. 2022 Jul 15;14(14):2909. doi: 10.3390/nu14142909 (PMC9322729; doi:10.3390/nu14142909)
Supplement: Supplementary file 1 [file nutrients-14-02909-s001.zip › nutrients-1792425-supplementary.pdf]

Table S1. Places from which the women bought or obtained food supplements reported by pregnant women in four European countries.

	**Finland**	**Italy**	**Poland**	**United Kingdom**	
**Place of purchase, n (%)**	*n=536*	*n=467*	*n=553*	*n=112*	***P*-value**[a]
Doctor, midwife, health visitor	14 (2.6)	13 (2.8)	23 (4.2)	19 (17.0)	*<0.001*
Pharmacy, without prescription	492 (91.8)	336 (72.0)	527 (95.3)	49 (43.8)	*<0.001*
Pharmacy, with prescription	16 (3.0)	161 (34.5)	117 (21.2)	6 (5.4)	*<0.001*
Supermarket / grocery store	123 (23.0)	15 (3.2)	8 (1.5)	49 (43.8)	*<0.001*
Health/herbal store	84 (15.7)	13 (2.8)	5 (0.9)	14 (12.5)	*<0.001*
Domestic online store	40 (7.5)	27 (5.8)	46 (8.3)	15 (13.4)	*0.288*
International online store	18 (3.4)	12 (2.6)	6 (1.1)	3 (2.7)	*0.558*
Network marketing (direct selling)	10 (1.9)	4 (0.9)	2 (0.4)	1 (0.9)	*0.582*
Other[b]	4 (0.7)	7 (1.5)	15 (2.7)	4 (3.6)	*0.252*

[a] Comparison among the countries, Chi-square test with Bonferroni correction.
[b] Gym or health spa, work, family, nutritionist, online pharmacy.

Table S2. Perceived recommendations for food supplement use before and during pregnancy and during breastfeeding as reported by pregnant women in four European countries.

	Finland	Italy	Poland	United Kingdom	
**Statement, n (%)**	*n=536*	*n=540*	*n=585*	*n=121*	***P-value*** [a]
**Multivitamin**					
Recommended before pregnancy	226 (42.2)	139 (25.3)	339 (57.7)	67 (55.4)	*<0.001*
Recommended during pregnancy	374 (69.8)	394 (71.6)	488 (83.0)	90 (74.4)	*<0.001*
Recommended during breastfeeding	256 (47.8)	166 (30.2)	364 (61.9)	57 (47.1)	*<0.001*
Not recommended	62 (11.6)	26 (4.7)	28 (4.8)	7 (5.8)	*<0.001*
**Vitamin A**					
Recommended before pregnancy	4 (0.8)	51 (9.3)	44 (7.5)	4 (3.9)	*<0.001*
Recommended during pregnancy	2 (0.4)	106 (19.3)	60 (10.2)	5 (4.1)	*<0.001*
Recommended during breastfeeding	3 (0.6)	49 (8.9)	35 (6.0)	3 (2.5)	*<0.001*
Not recommended	447 (83.4)	132 (24.0)	220 (37.4)	75 (62.0)	*<0.001*
**Folic acid**					
Recommended before pregnancy	455 (85.0)	472 (85.8)	544 (92.5)	88 (72.7)	*<0.001*
Recommended during pregnancy	441 (82.3)	429 (78.0)	504 (85.7)	82 (67.8)	*<0.001*
Recommended during breastfeeding	55 (10.3)	64 (11.6)	117 (19.9)	8 (6.6)	*<0.001*
Not recommended	16 (3.0)	1 (0.2)	5 (0.9)	2 (1.7)	*0.003*
**Vitamin D**					
Recommended before pregnancy	253 (47.2)	94 (17.1)	274 (46.6)	48 (39.7)	*<0.001*
Recommended during pregnancy	328 (61.2)	175 (31.8)	325 (55.3)	73 (60.3)	*<0.001*
Recommended during breastfeeding	255 (47.6)	119 (21.6)	250 (42.5)	50 (41.3)	*<0.001*
Not recommended	47 (8.8)	58 (10.6)	46 (7.8)	3 (2.5)	*0.180*
**Vitamin C**					
Recommended before pregnancy	50 (9.3)	108 (19.6)	143 (24.3)	18 (14.9)	*<0.001*
Recommended during pregnancy	56 (10.5)	163 (29.6)	132 (22.5)	25 (20.7)	*<0.001*
Recommended during breastfeeding	40 (7.5)	102 (18.6)	85 (14.5)	14 (11.6)	*<0.001*
Not recommended	207 (38.6)	74 (13.5)	139 (23.6)	29 (24.0)	*<0.001*
**Iodine**					
Recommended before pregnancy	12 (2.2)	33 (6.1)	134 (23.0)	3 (2.5)	*<0.001*
Recommended during pregnancy	24 (4.5)	76 (14.1)	253 (43.3)	5 (4.1)	*<0.001*
Recommended during breastfeeding	10 (1.9)	33 (6.1)	96 (16.4)	3 (2.5)	*<0.001*

Not recommended	239 (44.6)	116 (21.5)	76 (13.0)	33 (27.3)	*<0.001*
**Calcium**					
Recommended before pregnancy	69 (12.9)	72 (13.4)	123 (21.1)	15 (12.4)	*0.002*
Recommended during pregnancy	184 (34.3)	176 (32.7)	195 (33.4)	31 (25.6)	*1.000*
Recommended during breastfeeding	126 (23.5)	155 (28.8)	157 (26.9)	23 (19.0)	*0.396*
Not recommended	133 (24.8)	71 (13.2)	90 (15.4)	25 (20.7)	*<0.001*
**Iron**					
Recommended before pregnancy	26 (4.9)	85 (15.8)	131 (22.4)	22 (18.2)	*<0.001*
Recommended during pregnancy	347 (64.7)	343 (63.6)	340 (58.2)	51 (42.2)	*<0.001*
Recommended during breastfeeding	62 (11.6)	123 (22.8)	144 (24.7)	23 (19.0)	*<0.001*
Not recommended	90 (16.8)	37 (6.9)	59 (10.1)	19 (15.7)	*<0.001*
**Vitamin B12**					
Recommended before pregnancy	49 (9.1)	98 (17.8)	164 (27.9)	20 (16.5)	*<0.001*
Recommended during pregnancy	75 (14.0)	226 (41.1)	224 (38.1)	24 (19.8)	*<0.001*
Recommended during breastfeeding	35 (6.5)	93 (16.9)	110 (18.7)	12 (9.9)	*<0.001*
Not recommended	172 (32.1)	63 (11.5)	72 (12.2)	24 (19.8)	*<0.001*
**Vitamin B (other than B12 and folic acid)**					
Recommended before pregnancy	27 (5.0)	103 (18.7)	140 (23.8)	12 (9.9)	*<0.001*
Recommended during pregnancy	39 (7.3)	204 (37.1)	179 (30.4)	16 (13.2)	*<0.001*
Recommended during breastfeeding	16 (3.0)	87 (15.8)	89 (15.1)	8 (6.6)	*<0.001*
Not recommended	185 (34.5)	57 (10.4)	79 (13.4)	26 (21.5)	*<0.001*
**Vitamin E**					
Recommended before pregnancy	12 (2.2)	52 (9.5)	82 (14.0)	5 (4.1)	*<0.001*
Recommended during pregnancy	10 (1.9)	100 (18.2)	80 (13.6)	5 (4.1)	*<0.001*
Recommended during breastfeeding	9 (1.7)	60 (10.9)	51 (8.7)	3 (2.5)	*<0.001*
Not recommended	215 (40.1)	85 (15.5)	118 (20.1)	31 (25.6)	*<0.001*
**Zinc**					
Recommended before pregnancy	14 (2.6)	35 (6.5)	70 (12.0)	5 (4.1)	*<0.001*
Recommended during pregnancy	14 (2.6)	73 (13.5)	85 (14.6)	6 (5.0)	*<0.001*
Recommended during breastfeeding	11 (2.1)	31 (5.8)	47 (8.1)	3 (2.5)	*<0.001*
Not recommended	201 (37.5)	100 (18.6)	119 (20.4)	29 (24.0)	*<0.001*
**Selenium**					
Recommended before pregnancy	9 (1.7)	26 (4.8)	59 (10.1)	2 (1.7)	*<0.001*
Recommended during pregnancy	10 (1.9)	62 (11.5)	86 (14.7)	3 (2.5)	*<0.001*

Recommended during breastfeeding	8 (1.5)	26 (4.8)	45 (7.7)	2 (1.7)	*<0.001*
Not recommended	208 (38.8)	115 (21.3)	126 (21.6)	29 (24.0)	*<0.001*
**Chromium**					
Recommended before pregnancy	2 (0.4)	16 (3.0)	44 (7.5)	2 (1.7)	*<0.001*
Recommended during pregnancy	2 (0.4)	45 (8.4)	50 (8.6)	1 (0.8)	*<0.001*
Recommended during breastfeeding	2 (0.4)	18 (3.3)	28 (4.8)	2 (1.7)	*<0.001*
Not recommended	212 (39.6)	126 (23.4)	147 (25.2)	32 (26.5)	*<0.001*
**Magnesium**					
Recommended before pregnancy	25 (4.7)	99 (18.4)	190 (32.5)	5 (4.1)	*<0.001*
Recommended during pregnancy	89 (16.6)	263 (48.8)	323 (55.3)	13 (10.7)	*<0.001*
Recommended during breastfeeding	27 (5.0)	97 (18.0)	151 (25.9)	6 (5.0)	*<0.001*
Not recommended	167 (31.2)	45 (8.4)	65 (11.1)	25 (20.7)	*<0.001*
**Manganese**					
Recommended before pregnancy	2 (0.4)	28 (5.2)	43 (7.4)	1 (0.8)	*<0.001*
Recommended during pregnancy	2 (0.4)	66 (12.2)	50 (8.6)	2 (1.7)	*<0.001*
Recommended during breastfeeding	2 (0.4)	28 (5.2)	27 (4.6)	1 (0.8)	*<0.001*
Not recommended	210 (39.2)	91 (16.9)	130 (22.3)	31 (25.6)	*<0.001*

[a] Comparison between the countries, Chi-square test with Bonferroni correction.

Table S3. Non-pregnancy related health conditions of the pregnant women participating in the study.

	All	Finland	Italy	Poland	United Kingdom	
**Health conditions, n (%)**	*n=1762*	*n=536*	*n=539*	*n=569*	*n=118*	***P*-value**[a]
Diabetes [b]	17 (1.0)	7 (1.3)	5 (0.9)	5 (0.9)	0 (0.0)	*1.000*
Heart disease/ high blood pressure/ high cholesterol	38 (2.2)	18 (3.4)	6 (1.1)	13 (2.3)	1 (0.8)	*0.354*
Thyroid disease	274 (15.6)	52 (9.7)	70 (13.0)	142 (25.0)	10 (8.5)	*<0.001*
Intestinal health conditions [c]	221 (12.5)	81 (15.1)	73 (13.5)	43 (7.6)	24 (20.3)	*<0.001*
Allergy/ asthma	340 (19.3)	170 (31.7)	67 (12.4)	86 (15.1)	17 (14.4)	*<0.001*
Rheumatism/ arthritis	29 (1.6)	5 (0.9)	12 (2.2)	9 (1.6)	3 (2.5)	*1.000*
Migraine/ frequent headaches	273 (15.5)	119 (22.2)	52 (9.6)	90 (15.8)	12 (10.2)	*<0.001*
Chronic anxiety/ depression	107 (6.1)	49 (9.1)	27 (5.0)	16 (2.8)	15 (12.7)	*<0.001*

[a] Comparison between the countries, Chi-square test with Bonferroni correction.
[b] Type 1 or type 2 diabetes.
[c] Health conditions including celiac disease, inflammatory bowel disease, irritable bowel syndrome and/or food intolerance.

Table S4. Frequency of food supplement use during pregnancy as reported by pregnant women in four European countries.

		**Food supplement users**	**Frequency of using food supplements, *n (%)* of users**				
**Finland**	**Total n**	***n (%)* of participants**	7 days a week	5 - 6 days a week	3 - 4 days a week	1 - 2 days a week	Less than once a week
Prenatal multivitamin	529	432 (81.7)	356 (82.4)	41 (9.5)	23 (5.3)	0 (0.0)	12 (2.8)
Other multivitamin	524	160 (30.5)	108 (67.5)	10 (6.3)	14 (8.8)	8 (5.0)	20 (12.5)
Vitamin A	528	7 (1.3)	1 (14.3)	0 (0.0)	0 (0.0)	0 (0.0)	6 (85.7)
Folic acid	527	146 (27.7)	110 (75.3)	11 (7.5)	7 (4.8)	5 (3.4)	13 (8.9)
Vitamin B12	528	58 (11.0)	29 (50.0)	7 (12.1)	5 (8.6)	4 (6.9)	13 (22.4)
Other vitamin B's	526	39 (7.4)	23 (59.0)	1 (2.6)	4 (10.3)	0 (0.0)	11 (28.2)
Vitamin C	528	73 (13.8)	31 (42.5)	4 (5.5)	7 (9.6)	4 (5.5)	27 (37.0)
Vitamin D	529	202 (38.2)	130 (64.4)	17 (8.4)	18 (8.9)	10 (5.0)	27 (13.4)
Vitamin E	528	12 (2.3)	5 (41.7)	0 (0.0)	0 (0.0)	0 (0.0)	7 (58.3)
Iodine	529	14 (2.6)	3 (21.4)	1 (7.1)	0 (0.0)	1 (7.1)	9 (64.3)
Calcium	529	130 (24.6)	74 (56.9)	14 (10.8)	15 (11.5)	12 (9.2)	15 (11.5)
Iron	526	208 (39.5)	122 (58.7)	21 (10.1)	33 (15.9)	10 (4.8)	22 (10.6)
Zinc	528	51 (9.7)	18 (35.3)	2 (3.9)	5 (9.8)	5 (9.8)	21 (41.2)
Selenium	529	8 (1.5)	1 (12.5)	1 (12.5)	0 (0.0)	0 (0.0)	6 (75.0)
Magnesium	528	159 (30.1)	62 (39.0)	22 (13.8)	24 (15.1)	23 (14.5)	28 (17.6)
Manganese	528	5 (0.9)	0 (0.0)	1 (20.0)	0 (0.0)	0 (0.0)	4 (80.0)
Chromium	529	6 (1.1)	1 (16.7)	0 (0.0)	0 (0.0)	0 (0.0)	5 (83.3)

		**Food supplement users**	**Frequency of using food supplements, *n (%)* of users**				
**Italy**	**Total n**	***n (%)* of participants**	7 days a week	5 - 6 days a week	3 - 4 days a week	1 - 2 days a week	Less than once a week
Prenatal multivitamin	469	354 (75.5)	333 (94.1)	7 (2.0)	9 (2.5)	2 (0.6)	3 (0.8)
Other multivitamin	461	164 (35.6)	144 (87.8)	2 (1.2)	10 (6.1)	2 (1.2)	6 (3.7)
Vitamin A	460	40 (8.7)	25 (62.5)	1 (2.5)	4 (10.0)	3 (7.5)	7 (17.5)
Folic acid	464	232 (50.0)	214 (92.2)	3 (1.3)	6 (2.6)	4 (1.7)	5 (2.2)
Vitamin B12	459	60 (13.1)	43 (71.7)	1 (1.7)	5 (8.3)	4 (6.7)	7 (11.7)
Other vitamin B's	461	70 (15.2)	50 (71.4)	1 (1.4)	6 (8.6)	5 (7.1)	8 (11.4)
Vitamin C	458	57 (12.4)	34 (59.6)	3 (5.3)	7 (12.3)	5 (8.8)	8 (14.0)
Vitamin D	458	67 (14.6)	39 (58.2)	0 (0.0)	4 (6.0)	15 (22.4)	9 (13.4)
Vitamin E	456	31 (6.8)	19 (61.3)	0 (0.0)	1 (3.2)	4 (12.9)	7 (22.6)
Iodine	458	27 (5.9)	19 (70.4)	0 (0.0)	0 (0.0)	1 (3.7)	7 (25.9)
Calcium	457	33 (7.2)	21 (63.6)	1 (3.0)	3 (9.1)	2 (6.1)	6 (18.2)
Iron	461	148 (32.1)	130 (87.8)	5 (3.4)	4 (2.7)	5 (3.4)	4 (2.7)
Zinc	457	25 (5.5)	16 (64.0)	0 (0.0)	1 (4.0)	1 (4.0)	7 (28.0)
Selenium	459	22 (4.8)	14 (63.6)	0 (0.0)	0 (0.0)	1 (4.5)	7 (31.8)
Magnesium	459	127 (27.7)	82 (64.6)	4 (3.1)	19 (15.0)	11 (8.7)	11 (8.7)
Manganese	456	18 (3.9)	9 (50.0)	1 (5.6)	1 (5.6)	1 (5.6)	6 (33.3)
Chromium	459	15 (3.3)	7 (46.7)	0 (0.0)	1 (6.7)	1 (6.7)	6 (40.0)

		**Food supplement users**	**Frequency of using food supplements, *n (%)* of users**				
**Poland**	**Total n**	***n (%)* of participants**	7 days a week	5 - 6 days a week	3 - 4 days a week	1 - 2 days a week	Less than once a week
Prenatal multivitamin	555	504 (90.8)	474 (94.0)	12 (2.4)	12 (2.4)	2 (0.4)	4 (0.8)
Other multivitamin	551	200 (36.3)	177 (88.5)	9 (4.5)	7 (3.5)	3 (1.5)	4 (2.0)
Vitamin A	551	26 (4.7)	21 (80.8)	0 (0.0)	1 (3.8)	2 (7.7)	2 (7.7)
Folic acid	550	179 (32.5)	166 (92.7)	2 (1.1)	7 (3.9)	2 (1.1)	2 (1.1)
Vitamin B12	549	66 (12.0)	54 (81.8)	3 (4.5)	3 (4.5)	2 (3.0)	4 (6.1)
Other vitamin B's	550	39 (7.1)	23 (59.0)	1 (2.6)	4 (10.3)	0 (0.0)	11 (28.2)
Vitamin C	550	90 (16.4)	49 (54.4)	5 (5.6)	10 (11.1)	11 (12.2)	15 (16.7)
Vitamin D	550	111 (20.2)	90 (81.1)	2 (1.8)	11 (9.9)	4 (3.6)	4 (3.6)
Vitamin E	550	21 (3.8)	16 (76.2)	1 (4.8)	1 (4.8)	0 (0.0)	3 (14.3)
Iodine	550	66 (12.0)	61 (92.4)	3 (4.5)	1 (1.5)	0 (0.0)	1 (1.5)
Calcium	551	49 (8.9)	25 (51.0)	2 (4.1)	4 (8.2)	10 (20.4)	8 (16.3)
Iron	551	147 (26.7)	124 (84.4)	4 (2.7)	8 (5.4)	5 (3.4)	6 (4.1)
Zinc	551	18 (3.3)	13 (72.2)	0 (0.0)	1 (5.6)	2 (11.1)	2 (11.1)
Selenium	551	22 (4.0)	15 (68.2)	1 (4.5)	1 (4.5)	1 (4.5)	4 (18.2)
Magnesium	551	270 (49.0)	215 (79.6)	9 (3.3)	16 (5.9)	10 (3.7)	20 (7.4)
Manganese	550	13 (23.6)	11 (84.6)	0 (0.0)	0 (0.0)	0 (0.0)	2 (15.4)
Chromium	552	11 (2.0)	9 (81.8)	0 (0.0)	1 (9.1)	0 (0.0)	1 (9.1)

		**Food supplement users**	**Frequency of using food supplements, *n* (%) of users**				
**United Kingdom**	**Total n**	***n* (%) of participants**	7 days a week	5 - 6 days a week	3 - 4 days a week	1 - 2 days a week	Less than once a week
Prenatal multivitamin	112	97 (86.6)	76 (78.4)	10 (10.3)	4 (4.1)	6 (6.2)	1 (1.0)
Other multivitamin	111	28 (25.2)	21 (75.0)	2 (7.1)	2 (7.1)	0 (0.0)	3 (10.7)
Vitamin A	110	1 (0.9)	0 (0.0)	1 (100.0)	0 (0.0)	0 (0.0)	0 (0.0)
Folic acid	111	10 (9.0)	7 (70.0)	1 (10.0)	1 (10.0)	0 (0.0)	1 (10.0)
Vitamin B12	111	9 (8.1)	6 (66.7)	0 (0.0)	1 (11.1)	1 (11.1)	1 (11.1)
Other vitamin B's	111	7 (6.3)	4 (57.1)	1 (14.3)	0 (0.0)	1 (14.3)	1 (14.3)
Vitamin C	111	6 (5.4)	3 (50.0)	2 (33.3)	1 (16.7)	0 (0.0)	0 (0.0)
Vitamin D	111	20 (18.0)	11 (55.0)	2 (10.0)	3 (15.0)	3 (15.0)	0 (0.0)
Vitamin E	111	2 (1.8)	1 (50.0)	0 (0.0)	1 (50.0)	0 (0.0)	0 (0.0)
Iodine	111	2 (1.8)	1 (50.0)	0 (0.0)	0 (0.0)	0 (0.0)	1 (50.0)
Calcium	112	7 (6.3)	4 (57.1)	2 (28.6)	1 (14.3)	0 (0.0)	0 (0.0)
Iron	112	21 (18.8)	12 (57.1)	4 (19.0)	1 (4.8)	4 (19.0)	0 (0.0)
Zinc	110	2 (1.8)	1 (50.0)	1 (50.0)	0 (0.0)	0 (0.0)	0 (0.0)
Selenium	111	2 (1.8)	1 (50.0)	0 (0.0)	1 (50.0)	0 (0.0)	0 (0.0)
Magnesium	111	2 (1.8)	2 (100.0)	0 (0.0)	0 (0.0)	0 (0.0)	0 (0.0)
Manganese	112	10 (8.9)	4 (40.0)	1 (10.0)	1 (10.0)	1 (10.0)	0 (0.0)
Chromium	111	1 (0.9)	0 (0.0)	1 (100.0)	0 (0.0)	0 (0.0)	0 (0.0)

Table S5. Daily intakes of nutrients from food supplements with no recommended intake levels during pregnancy.

	Finland	Italy	Poland	United Kingdom	
**Nutrient**	*n=470*	*n=360*	*n=439*	*n=97*	***P*-value**
**Betacarotene, μg/d**					
User, n (%)	3 (0.6)	99 (27.5)	76 (17.3)	1 (1.0)	*<0.001* [a]
Dose, median (IQR)	1200	3000 (1800 – 3000)	3000 (1929 – 3000)	80.1	*0.100* [b]
Dose, range	400 - 2400	200 - 6000	180 - 6000	-	
**Vitamin B1 (thiamin), mg/d**					
User, n (%)	394 (83.8)	271 (75.3)	124 (28.3)	77 (79.4)	*<0.001* [a]
Dose, median (IQR)	5.0 (2.6 – 5.0)	1.1 (1.1 – 1.4)	1.5 (1.3 – 1.5)	3.0 (1.4 – 3.0)	*<0.001* [b]
Dose, range	0.37 – 55.0	0.5 – 26.2	0.4 – 9.0	1.1 – 8.0	
**Vitamin B2 (riboflavin), mg/d**					
User, n (%)	393 (83.6)	274 (76.1)	116 (26.4)	77 (79.4)	*<0.001* [a]
Dose, median (IQR)	5.0 (2.8 – 5.0)	1.5 (1.4 – 1.7)	1.6 (1.6 – 1.6)	2.0 (1.4 – 2.0)	*<0.001* [b]
Dose, range	0.5 – 55.0	0.6 – 15.0	0.7 – 20.0	0.9 – 8.0	
**Vitamin B3 (niacin), mg/d**					
User, n (%)	390 (83.0)	269 (74.7)	119 (27.1)	65 (67.0)	*<0.001* [a]
Dose, median (IQR)	20.0 (17.0 – 20.0)	16.0 (16.0 – 22.0)	18.0 (16.0 – 20.0)	20.0 (20.0 – 20.0)	*<0.001* [b]
Dose, range	4.5 – 65.0	6.9 – 51.0	7.5 – 120.0	2.1 – 32.0	
**Vitamin B5 (pantothenic acid), mg/d**					
User, n (%)	390 (83.0)	268 (74.4)	120 (27.3)	76 (78.4)	*<0.001* [a]
Dose, median (IQR)	5.0 (5.0 – 5.8)	6.0 (6.0 – 6.0)	10.0 (6.0 – 10.0)	6.0 (6.0 – 6.0)	*<0.001* [b]

Dose, range	1.8 – 55.0	2.6 – 25.0	3.0 – 150.0	2.6 – 20.0	
**Vitamin B7 (biotin), µg/d**					
User, n (%)	343 (73.0)	263 (73.1)	105 (23.9)	76 (78.4)	*<0.001* [a]
Dose, median (IQR)	30.0 (30.0 – 30.0)	50.0 (35 – 60.0)	100.0 (60.0 – 100.0)	150.0 (50.0 – 150.0)	*<0.001* [b]
Dose, range	5.0 – 10000	15.0 – 525.0	3.0 – 500.0	12.0 – 225.0	
**Vitamin B12 (cobalamin), µg/d**					
User, n (%)	402 (85.5)	278 (77.2)	266 (60.6)	82 (84.5)	*<0.001* [a]
Dose, median (IQR)	3.0 (3.0 – 3.0)	2.5 (2.5 – 2.6)	3.5 (2.6 – 4.0)	6.0 (2.6 – 6.0)	*<0.001* [b]
Dose, range	0.3 – 1013.5	0.4 – 285.7	0.5 – 504.0	1.5 – 500.0	
**Vitamin C, mg/d**					
User, n (%)	397 (84.5)	192 (53.3)	148 (33.7)	84 (86.6)	*<0.001* [a]
Dose, median (IQR)	120.0 (102.9 – 120.0)	100.0 (80.0 – 100.0)	120.0 (80.0 – 180.0)	70.0 (70.0 – 80.0)	*<0.001* [b]
Dose, range	7.5 – 1082.0	10.0 – 2000.0	20.0 – 1110.0	30.0 – 200.0	
**Vitamin K, µg/d**					
User, n (%)	66 (14.0)	17 (4.7)	6 (1.4)	73 (75.3)	*<0.001* [a]
Dose, median (IQR)	90.0 (75.0 – 90.0)	35.0 (35.0 – 44.0)	100.0 (55.0 – 150.0)	70.0 (70.0 – 70.0)	*<0.001* [b]
Dose, range	25.0 – 90.0	21.4 – 140.0	40.0 – 300.0	20.0 – 150.0	
**Iron, mg/d**					
User, n (%)	406 (86.4)	293 (81.4)	349 (79.5)	79 (81.4)	*0.270* [a]
Dose, median (IQR)	20.0 (20.0 – 70.0)	30.0 (27.0 – 41.1)	30.0 (27.0 – 60.0)	17.0 (17.0 – 17.0)	*<0.001* [b]
Dose, range	2.7 – 220.0	2.1 – 210.0	7.5 – 260.0	7.3 – 158.0	
**Manganese, mg/d**					
User, n (%)	85 (18.1)	146 (40.6)	89 (20.3)	4 (4.1)	*<0.001* [a]

Dose, median (IQR)	1.8 (1.7 – 1.8)	2.0 (2.0 – 2.0)	1.0 (1.0 – 1.0)	0.6 (0.3 – 0.9)	*<0.001* [b]
Dose, range	0.4 – 2.5	0.5 – 10.0	0.4 – 5.0	0.3 – 1.0	
**Chromium, μg/d**					
User, n (%)	82 (17.4)	80 (22.2)	2 (0.5)	18 (18.6)	*<0.001* [a]
Dose, median (IQR)	25.0 (25.0 – 25.4)	40.0 (40.0 – 40.0)	85.0	40.0 (40.0 – 40.0)	*<0.001* [b]
Dose, range	12.5 – 50.0	10.0 – 60.0	50.0 – 120.0	4.5 – 150.0	
**Copper, mg/d**					
User, n (%)	261 (55.5)	207 (57.5)	96 (21.9)	75 (77.3)	*<0.001* [a]
Dose, median (IQR)	1.0 (1.0 – 1.0)	1.2 (0.5 – 1.2)	1.0 (1.0 – 1.0)	1.0 (1.0 – 1.0)	*<0.001* [b]
Dose, range	0.3 – 1.5	0.1 – 2.6	0.4 – 1.0	0.4 – 2.5	

[a] Comparison between the countries, Chi-square test with Bonferroni correction.
[b] Comparison between the countries, Kruskal-Wallis test with Bonferroni correction.
